# Surfing Time–Motion Characteristics Possible to Gain Using Global Navigation Satellite Systems: A Systematic Review

**DOI:** 10.3390/s24113455

**Published:** 2024-05-27

**Authors:** Gaizka Mejuto, Carlos David Gómez-Carmona, Jokin Gracia, Markel Rico-González

**Affiliations:** 1 Department of Didactics of Musical, Plastic and Corporal Expression, University of the Basque Country, UPV-EHU, 48940 Leioa, Spain; gaizka.mejuto@ehu.eus; 2 Biovetmed & Sportsci Research Group, Department of Physical Activity and Sport, Faculty of Sport Sciences, University of Murcia, 30720 San Javier, Spain; 3 Optimization of Training and Sports Performance Research Group, Department of Didactics of Music, Plastic and Body Expression, Faculty of Sport Sciences, University of Extremadura, 10003 Caceres, Spain; 4 Department of Strength and Conditioning, Athletic Club of Bilbao, 48196 Bilbao, Spain; jokingracia@hotmail.com

**Keywords:** global positioning systems, surf, motion, tracking, sport, sensors

## Abstract

The popularity of surfing has increased exponentially, reaching its recent debut in the Olympic Games. However, surfing suffers from a relative immature technological market, while in other sports some technologies such as global navigation satellite systems (GNSSs) have become an essential work material for strength and conditioning and head coaches. This article aims to systematically review surfers’ time–motion demands based on GNSSs. A systematic review of relevant articles was carried out using five main databases (PubMed, ProQuest Central, SCOPUS, SPORTDiscus, and FECYT (Web of Sciences, CCC, CIDW, KJD, MEDLINE, RSCI, and SCIELO)) until 23 March 2024. From the 238 studies initially found, 9 were included in the qualitative synthesis. In these, GNSS devices were employed with male (n = 143) and female (n = 28) surfers from different levels during competition and training situations. The studies show that the intermittent nature of the sport is evident, with substantial periods spent paddling and waiting punctuated by relatively brief high-intensity efforts when riding waves at high speeds. Notable differences emerged between competition and training demands, suggesting potential mismatches in how athletes currently prepare compared to event requirements. These novel insights allow quantifying surfing’s harsh physiological requirements and could guide conditioning practices to better meet the sport’s unique characteristics across populations. Therefore, training should emulate the lengthy aerobic capabilities needed for the paddling volumes observed, while also targeting the anaerobic systems to meet the repeated high-intensity surf riding efforts. However, inconsistencies in methods and reporting practices limit direct comparisons and comprehensive profiling of the sport’s physical characteristics.

## 1. Introduction

Being one of the oldest sports in modern history [1], surfing is becoming a popular water sport [2]. Currently, participation in surfing has increased worldwide, reaching 35 million people practicing surfing [1]. In 2020, surfing debuted in the Olympic Games of Tokyo [2], leading to athletes pursuing a professional career [2].

For a better understanding of surfing competition and its physical demands, surfing is based on elimination heats of 20–40 min, where a judge provides a score to the 2–4 surfers taking part in the heat. The judge provides their score based on the variety of maneuvers and performance on the most critical part of the wave. Mainly, a panel of five judges will score each ride based on the following “judging criteria”: commitment and degree of difficulty, innovative and progressive maneuvers, combinations of major maneuvers, variety of maneuvers, speed, power, and flow [1]. The athletes that achieve more points will advance to the next round, while the others are eliminated [2]. Although prior research demonstrates that surfers are not frequently injured (0.74–1.79 per 1000 h of surfing) [3], these characteristics lead this intermittent activity to request from athletes a great strength, power, and endurance of the trunk, abdominal musculature, and upper and lower limbs [4]. These areas of physical fitness could help surfers during the aforementioned maneuvers performed in an unpredictable environmental factor (e.g., waves’ behavior) [5]. These requirements make surfing a demanding sport that requires a high level of training. The athlete’s optimal physical fitness is more difficult to achieve when knowing that the environmental conditions dictate whether suitable sized waves and swell allow for viable surfing conditions and, subsequently, the availability of training sessions. In fact, prolonged ocean swells caused by distant storms last between 2 and 6 days, lending surfers sporadic and brief opportunities to spend up to 4 h in the water [3]. 

At a time when coaches based their training prescriptions on their great understanding of the physical demands, technological manufacturers developed and adapted their devices to facilitate and improve work in some sectors, where sport was not an exception [6,7,8]. However, although there has been an increment in the popularity of water sports, surfing in particular has been raising attention to its yet immature technology market [9]. While several available solutions (e.g., video recordings) aim to characterize surf session events, there are other technologies that have helped in improving athletes’ performance in other sports such as global navigation satellite systems (GNSSs). 

In the past century, GNSSs have started to be used with military aims [7]. However, although most of them continue with this use (i.e., GPS, GLONASS, and Beidou), these devices have been incorporated into several sport disciplines, providing a wide range of external workload variables that allow coaches to objectively understand what is happening during competition [10,11,12,13]. GNSSs are a technology composed by a reference surface (i.e., the constellations and their satellites) and receivers (i.e., GNSSs). In this way, a GNSS receptor, located in the athlete’s upper back, receives a signal of at least four satellites from GLONASS, BeiDou, Galileo, etc., which through trigonometry calculate the positioning of the receptor (the athlete’s positioning) [14]. This information depends on different important factors such as the rate/frequency or dilution of precision (DOP). When recording the positioning during a certain period, the raw information can be used by team staff to analyze surfers’ performance based on a wide range of variables such as distance-based variables (e.g., total distance, distance at different intensities) or speed-based variables (e.g., maximum speed reached). These variables, and subsequently this systematic review, could help coaches and surfers to understand what is happening during training and competition, which could provide a great opportunity for achieving a peak performance. 

In this way, several systematic reviews have been performed for highlighting the reference values of athletes’ physical fitness during training and competition in other sports [15,16,17,18]. However, to the best of the authors’ knowledge, no systematic review has been published about the time–motion demands based on GNSS objective measures in surfing. Hence, this article aims to systematically review surfers’ physical fitness demands based on GNSS data. The authors hope this systematic review provides a mark of reference that could help surfers and coaches during training prescription. 

## 2. Materials and Methods

### 2.1. Experimental Approach to the Problem

Two guidelines were considered for running out this systematic review:

- The Preferred Reporting Items for Systematic Reviews and Meta-Analyses (PRISMA) guidelines [19].
- Guidelines for performing systematic reviews in sport sciences [20].

### 2.2. Information Sources

A systematic search of five databases (PubMed, ProQuest Central, FECYT (Web of Sciences, CCC, CIDW, KJD, MEDLINE, RSCI, and SCIELO), SCOPUS, and SPORTDiscus) was performed to identify articles published prior to 25 March 2024. 

### 2.3. Search Strategy

As stated by PRISMA, the PICO strategy was followed for performing the search. In this way, the authors could ensure that an explicit statement of the question was designed based on the population, intervention, comparison, and outcomes. Hence, the search strategy used was as follows:

(surfing OR surfer OR surfboard) AND (“global positioning system*” OR GPS OR “Global Navigation Satellite System*” OR GNSS)

During the search process, the authors of this systematic review were not blinded to the authors’ names or publication sources’ title. When possible, the filter “scientific articles” was selected in databases. 

### 2.4. Eligibility Criteria and Data Extraction

Once the articles’ information (database, title, authors, journal, and publication date) was downloaded and transferred into an Excel spreadsheet (Microsoft Corporation, Redmond, WA, USA), those articles that appeared in more than one database were detected and identified as duplicates. Then, all articles’ title, abstract, and if necessary, full text were checked and identified following the inclusion/exclusion criteria highlighted in Table 1. 

Once two authors completed the articles’ identification independently, all results were compared. If any disagreement appeared, it was solved by revising the articles with the two authors that completed the previous step. All records were stored in the spreadsheet.

### 2.5. Assessment of Study Methodology

As a methodological index for non-randomized studies, the MINORS scale was used. The MINORS scale is a list that contains 8 essential points and it is expanded to 12 points when the studies to be treated are comparative. In this case, it was assessed considering 9 items (out of 18 points) due to the non-possibility to apply (NA) 3 of them. The score that each section receives can be from 0 to 2, depending on the quality obtained by each point. The MINORS checklist asks the following information (2 = High quality; 1 = Medium quality; 0 = Low quality).

## 3. Results

### 3.1. Identification and Selection of Studies

**Figure 1:**
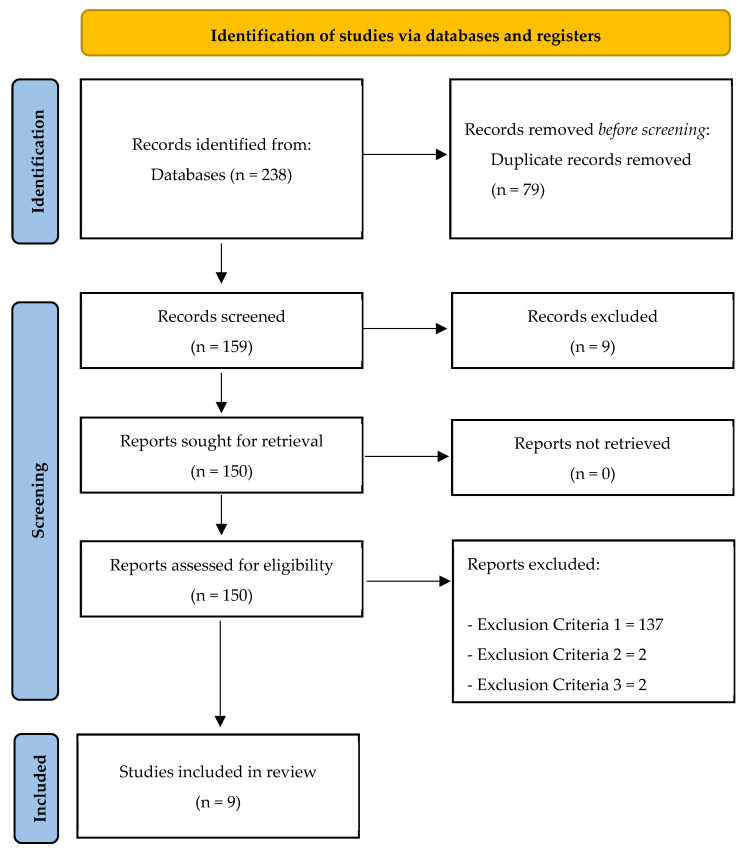
Flow diagram of the study.

There was a total of 238 (PubMed = 16; ProQuest Central = 29; FECYT = 122; SCOPUS = 55; SPORTDiscus = 16) original articles, of which 79 were duplicates. Thus, a total of 159 unique articles were identified. After checking titles and abstracts, nine articles were excluded because they did not meet inclusion criteria number five. The full text of the remaining 150 articles was then analyzed; 137 articles were excluded because they did not meet inclusion criteria number one, 2 articles were excluded because they did not meet inclusion criteria number two, and 2 articles were excluded because they did not meet exclusion criteria number three. Thus, a total of 9 articles met all the inclusion criteria and were included in the final qualitative synthesis (Figure 1).

### 3.2. Quality Assessment

The quality assessment for this systematic review can be found in Table 2. The methodological quality of the included studies was assessed using the methodological index for non-randomized studies (MINORS) criteria. All nine studies scored 20 out of 24 possible points, indicating a high overall methodological rigor. Across the studies, the aims were clearly defined, patient selection and inclusion was appropriate, assessments were adjusted to meet the stated objectives, and the follow-up procedures were consistent with the study goals, all receiving maximum scores. However, none of the studies reported dropout rates below 5% or provided prospective estimations of required sample sizes, resulting in scores of zero for those items. The criteria related to the inclusion of control groups and group comparisons were not applicable given the likely descriptive nature of the studies. Appropriate statistical analyses were conducted in all studies based on maximum scoring for that item. While the studies demonstrated strengths in defining objectives, participant recruitment, data collection, and analyses, there were shortcomings in reporting attrition rates and justifying sample sizes a priori.

### 3.3. Study Characteristics

A total of nine studies have been included in the qualitative synthesis. In these studies, a total of 22 females (England) and 73 males (Spain, New Zealand, and Australia) took part in studies where time–motion was analyzed during competition, while 6 females (Portugal) and 70 males (Australia, Portugal, and the United Kingdom) took part in studies where time–motion was analyzed during training sessions. 

The used GNSSs were as follows: Catapult S5, Catapult Sports, Melbourne, Australia, Polar Electro V800; Polar Inc., Kempele, Finland; GPSports Team AMS v1.6.3.0, Canberra, Australia; SurfTraX, Southport, Australia; G3 GPS monitor (Polar Electro, Oy, Helsinki, Finland); Polar V800, Polar Electro, Kempele, Finland; GPSports HPISPU; and VX Sport VX110 Log, Visuallex Sport International Ltd., Lower Hutt, New Zealand, with a rate/frequency between 1 and 15 Hz. The horizontal dilution of precision and the number of satellites connected were not detailed in most of the articles. 

Regarding the reference competition values, it is not easy to establish a range in most of them due to different disparities in their description (different levels of competition, different duration of recording time, or different units to describe the time motion). The detailed characteristics of studies were extracted and clustered into Table 3 and Table 4.

## 4. Discussion

The ability to objectively monitor the physical demands placed on athletes during training and competition is crucial for optimal performance preparation and injury prevention [30]. While various technologies like video analysis and athlete self-reporting have been utilized in surfing, the application of global navigation satellite systems (GNSSs) offers a more comprehensive and objective assessment of the external workloads experienced by surfers [6,7,8]. Although surfing’s popularity has increased substantially, reaching 35 million practitioners worldwide [1], there is limited research quantifying key performance analytics for surfers using technologies common in other sports [2]. Therefore, this systematic review aimed to synthesize the existing literature on the use of GNSS technology to quantify the time–motion demands of recreational, amateur, and professional surfers during training and competition.

The main findings indicate that while only a limited number of studies (n = 9) have employed GNSS monitoring in surfing populations to date [21,22,23,24,25,26,27,28,29], these investigations provide valuable insights into the external loads that surfers are exposed to. The studies included both male (n = 143) [22,23,24,25,26,27,28,29] and female (n = 28) [21,27] surfers across the professional [21,26], national [23,24], amateur [29], junior [27], and recreational [22,28] levels. A variety of GNSS devices were utilized including Catapult [21], Polar [22,23,27], GPSports [24,28], VX Sport [29], and custom-made SurfTraX [25,26] units operating at rates/frequencies between 1 and 15 Hz. Key variables extracted focused on quantifying distances covered, speeds attained, durations, and activity profiles during surfing sessions and competitive events.

### 4.1. External Workload Demands Profile in Surfing

The GNSS data from the studies included in this review allow us to characterize the external workload demands profile of surfing in a comprehensive manner. In terms of total distances covered, competitive surfing heats of 20–40 min saw elite males cover around 1200–2000 m [21,24,25], with recreational male surfers averaging nearly 4000 m per hour of water time [22]. Over longer durations, amateur males covered approximately 5000 to 6000 m across a 2 h surf training session [28,29].

A substantial portion of this overall distance was accrued while paddling between waves, which accounted for around 650–1000 m during competition for professionals [26]. Paddling represented 42.6% of time for amateurs over 2 h [29], and 47% over recreational sessions lasting around 1 h [22]. Time spent stationary while waiting for acceptable waves was also high at 52.8% for amateurs [29] and 41.8% for recreational surfers [22].

The much shorter periods spent actually riding waves highlight the intermittent nature of the sport’s external demands. Wave riding constituted just 2.5% of time for amateurs [29] and 8.1% for recreational surfers [22]. The number of wave rides completed ranged from 3 to 10 per 20 min heat for elite competitors [21,26]. Wave riding distances were generally between 24 and 117 m [26], with maximum distances up to 155 m for elite females [21] and 180 m for elite males [25].

The peak speeds attained while wave riding were substantial, reaching 40 km/h for top professionals [24] and around 20–25 km/h [22,27] to 30 km/h [28] for amateurs and recreational participants [14,19,20]. However, the average wave riding speeds range from 5 to 15 km/h, indicating the importance of intermittent high-intensity efforts [24,25]. Overall, these data showcase the harsh demands of surfing, with lengthy periods of paddling and waiting punctuated by explosive bouts when riding waves at high speeds over relatively short distances.

### 4.2. Competition vs. Training Demands

When examining the GNSS variables between the competition and training scenarios, some notable differences emerge that may indicate discrepancies in the external workload demands. The total covered distance in training is higher (4000 to 6000 m) during 2-h sessions [28,29] in comparison with competitive 20–40 min heats with lower total distances (1200 to 2000 m) [21,24,25]. In contrast, the high-intensity nature of the wave riding efforts appears more pronounced during competition (peak wave speed: 40 km/h; riding distance: 155 to 180 m; 10 rides per 20 min = 30 rides per hour) [21,24,25,26] in comparison with training sessions (peak wave speed: 20–30 km/h; riding distance: 24-117 m; 20 rides per hour) [22,27,28,29].

The contrasting profiles point to competition perhaps requiring higher-intensity efforts but less overall volume, while training may emphasize building aerobic capacity through the greater total distances covered. In considering these findings, it should be noted that the training data came primarily from recreational and amateur populations rather than direct practice sessions for elite competitors. As such, there may be limitations in how representative these training demands are of how professional surfers actually prepare. Additionally, the competitive loads were only captured during short heats rather than full multi-day events which likely require supplementary training to meet the overall volume demands. Future research directly comparing world-class surfers’ training vs. competition demands within the same study would help to clarify whether the current practices are optimally structured.

### 4.3. Technical Features of GNSS Devices

Key technical factors impacting GNSS data validity and reliability include the rate/frequency, number of satellite receivers, dilution of precision (DOP), and device body placement. Regarding the rate/frequency, studies ranged from 1 [22], 2.4 [27], and 4 Hz [29] to 10 [21,25,26] or 15 Hz [28]. Higher frequencies, if the accuracy of the device is at the same level or a better level, better capture the rapid movements inherent to surfing performance [6,7]. Therefore, the appropriate recording rate is a trade-off based on the specific activities being measured and the duration of data collection required, being considered as less than 10 Hz in team sports [31].

Another important device specification is the number of satellite receivers being maintained and the horizontal dilution of precision (HDOP) [6]. Most studies did not report this information, but one noted maintaining receptions with 13 ± 1 satellites [21] and another >8 satellites [23]. Having more satellite links provides q better signal reception to enhance positional accuracy [32]. As well as this, HDOP metric accounts for the geometric strengths of the satellite configurations and was only reported at excellent values of less than 1.0 in a couple of studies [21,26]. Lower HDOP values indicate better positional accuracy [33].

Finally, inconsistent body placement for wearing the GNSS devices emerged across the studies. Devices were placed at the upper back near the spine and between the shoulder blades [21,24,28], on the wrist [23], or around the biceps [25,26]. Previous research identified the upper back as the best location for detecting position coordinates by GNSSs [10], and needs to be attached with an fixed vest to ensure no-movement during the assessments [34]. The placement on the wrist or biceps is not recommended due to the trunk acting as a screen, not allowing the GNSS signal reception, and potentially increasing the measurement error due to the increased separation from the body’s main inertial forces during movement [35].

Overall, there are clear trade-offs in the technical specifications that researchers must balance based on study aims and logistics. Higher sampling rates (at least 10 Hz) and maintaining receptions with more satellites (>12) and with lower HDOP values (<1.0) should theoretically yield more precise data for capturing surfing’s high-intensity, multi-directional movement patterns. However, factors like body-worn placement and environmental conditions are additional sources of uncertainty. Consistent body placements at the upper back with fitted vests that ensure no movement could help to improve assessments. Transparently reporting these details allows more rigorous analysis of the strengths and limitations inherent to a study’s data capture approach.

### 4.4. Limitations and Future Research Directions

While this systematic review provides novel insights into the external workload demands of surfing across levels and scenarios, there are some inherent limitations that should be acknowledged. Firstly, the total number of studies meeting the inclusion criteria was relatively low (n = 9), indicating this is still an emerging area of research focus. The sample sizes within the individual studies were also generally small, albeit a result of the practical constraints of data collection in field environments. There was also a lack of consistency in the ways external load variables were quantified and reported across studies. Some provided means/peaks, while others only gave ranges. Several did not disclose critical device specifications like sampling rates and satellite receivers that impact data accuracy. These inconsistencies limit the ability to directly compare and synthesize the findings. Additionally, most studies examined male participants, with only one providing gender-based comparisons. There was also an underrepresentation of data on the highest elite professional competitive levels. The external validity of the identified demands may not extend across all surfing contexts and populations.

To address these limitations, future research should prioritize standardizing methods and reporting practices to improve the comparability of results across studies. Achieving a consensus on the key variables and metrics to monitor would facilitate a more comprehensive profiling of external loads. Further work directly comparing demands between genders, ability levels, and training versus competition within consistent experimental designs is warranted. Evaluating if current training practices adequately prepare surfers for the demands of peak competitive performances should also be a focus. Technological advances could aid data capture, with integrated video-based validation to quantify measurement errors. Exploring applications of machine learning and wearable textiles may enhance the modeling of complex multi-directional surf movements. 

## 5. Conclusions

This systematic review highlights the limited but growing body of research utilizing GNSS technology to objectively quantify the external workload demands of surfing across various populations and scenarios. The intermittent nature of the sport is evident, with substantial periods spent paddling and waiting punctuated by relatively brief high-intensity efforts when riding waves at high speeds. Notable differences emerged between competition and training demands, suggesting potential mismatches in how athletes currently prepare compared to event requirements. However, inconsistencies in methods and reporting practices limit direct comparisons and comprehensive profiling of the sport’s physical characteristics.

The findings provide an initial evidence-based framework to guide conditioning practices aimed at developing the specific physical capacities required for surfing performance. Training should emulate the lengthy aerobic capabilities needed for the paddling volumes observed, while also targeting the anaerobic systems to meet the repeated high-intensity surf riding efforts. An increased emphasis on quantifying external loads during elite professionals’ actual training could identify potential deficiencies in the current approaches. Standardizing key load monitoring metrics and methods is recommended to facilitate more rigorous research that enhances the understanding and preparation of these unique athletes across levels. Emerging technologies may aid in the modeling of surfing’s complex multi-directional movements.

## Figures and Tables

**Table 1:** Inclusion/exclusion criteria.

Item	Inclusion	Exclusion
Population	Surfer athletes	Non-surfer athletes
Intervention or Exposure	Athletes surfing during training and/or competition	Surfers training out of the water
Comparison	Not applicable	Not applicable
Outcome[s]	Articles that contemplate results in populations related to surfing demands measured through a GNSS	Articles that do not contemplate results in populations related to surfing demands or measured without a GNSS
Other criteria	Peer reviewed, original, full-text studies written in English or Spanish	Written in another language or non-peer-reviewed original full-text studies

**Table 2:** Methodological assessment of the included studies.

Reference	1	2	3	4	5	6	7	8	9	10	11	12	Score
Barlow et al. [21]	2	2	2	2	2	2	0	0	NA	NA	NA	2	20/24
Barlow et al. [22]	2	2	2	2	2	2	0	0	NA	NA	NA	2	20/24
Fernandez-Gamboa et al. [23]	2	2	2	2	2	2	0	0	NA	NA	NA	2	20/24
Farley Et al. [24]	2	2	2	2	2	2	0	0	NA	NA	NA	2	20/24
Farley et al. [25]	2	2	2	2	2	2	0	0	NA	NA	NA	2	20/24
Farley et al. [26]	2	2	2	2	2	2	0	0	NA	NA	NA	2	20/24
Silva et al. [27]	2	2	2	2	2	2	0	0	NA	NA	NA	2	20/24
O’Neill et al. [28]	2	2	2	2	2	2	0	0	NA	NA	NA	2	20/24
Secomb et al. [29]	2	2	2	2	2	2	0	0	NA	NA	NA	2	20/24

Note: NA = not applicable. The MINORS checklist (2 = High quality; 1 = Medium quality; 0 = Low quality): Clearly defined objective (Item 1); Inclusion of patients consecutively (Item 2); Information collected retrospectively (Item 3); Assessments adjusted to objective (Item 4); Evaluations carried out in a neutral way (Item 5); Follow-up phase consistent with the objective (Item 6); Dropout rate during follow-up less than 5% (Item 7); Prospective estimation of sample size (Item 8); Adequate control group (Item 9); Simultaneous groups (Item 10); Homogeneous starting groups (Item 11); Appropriate statistical analysis (Item 12).

**Table 3:** Surfing demands during competition.

Ref.	Sample				Device’s Characteristics				Results	
	Participants	Level	Competition	Duration	Device Model	Hz	Number of Satellites Used	Place	Variable	Value
Barlow et al. [21]	N: 22 (age: 20.49 ± 5.32 y; Stature: 165.2 ± 4.8 cm; Weight: 63.0 ± 6.8 kg) S: Female C: England	PRO	United Kingdom Professional Surfing Association events	ND	Catapult S5, Catapult Sports, Australia	10	11 to 15 (mean = 13 ± 1)	Inside two knotted nitrile gloves in order to waterproof the unit and then located inside the wetsuit between the shoulder blades in-line with the spine.	Number or rides	7 ± 3
									Mean max ride speed (m/s)	6.55 ± 0.97
									SD of ride speeds	1.30 ± 0.70
									Mean ride time (s)	18.1 ± 12.64
									Max ride time (s)	32.07 ± 22.85
									Min ride time (s)	6.69 ± 3.15
									SD of ride times (s)	12.21 ± 16.00
									Mean ride distance (m)	78.12 ± 80.02
									Max ride distance (m)	155.93 ± 196.14
									Min ride distance (m)	24.17 ± 15.07
									SD of ride distance	56.47 ± 99.80
									Total time spent riding (s)	114.52 ± 73.73
									Distance surfing (m)	488 01 ± 434.84
									Total distance (m)	1267.43 ± 579.49
									Distance surfing (%)	35.60 ± 13.44
									Time sitting (%)	62.58 ± 10.18
									Time padding (%)	30.70 ± 9.44
									Time riding (%)	6.73 ± 2.91
Fernandez-Gamboa et al. [23]	N: 10 (age: 28.50 ± 11.09 y; Stature: 177.10 ± 5.54 cm; Weight: 70.20 ± 5.49 kg S: Male C: Spain	National-level open division	“Euskaltel Euskal Zirkuitua” championship	A competition heat	Polar Electro V800; Polar Inc., Kempele, Finland	2.4	>8	Attached to the wrist of the participants and outside the wetsuit	Total distance (m)	447.51 ± 126.31 (min: 243.90; max: 609.70)
									Padding distance (m)	353.66 ± 149.28 (min: 243.90; max: 550.10)
									Wave riding distance (m)	93.85 ± 84.26 (min: 12.90; max: 278.90)
									Wave riding duration	3.13 ± 2.35 (min: 0.95; max: 8.24)
									Stationary time	59.62 ± 13.09 (min: 38.64; max: 78.00)
									Active time (%)	40.17 ± 13.37 (min: 20.20; max: 61.32)
									Wave riding peak velocity (m/s^−1^)	0.61 ± 0.25 (min: 0.25; max: 1.38)
									Wave riding mean velocity (m/s^−1^)	0.50 ± 0.26 (min: 0.16; max: 1.31)
Farley et al. [24]	N: 12 (age: 23.6 ± 5.7 y; Stature: 179.2 ± 6.8 cm; Weight: 73 ± 10.3 kg) S: Male C: New Zealand	Ranked in the national top 30	New Zealand Surf Association competition	2 competitions (20 min heats)	GPSports Team AMS v1.6.3.0, Australia	ND	ND	Water tight sealed bag, turned on to record, then positioned under the wet suit of the subject around the upper thoracic vertebra and scapula	Average speed (km/h)	3.7 ± 0.6
									Max speed (km/h)	33.4 ± 6.5
									Max speed recorded (km/h)	45
									Total distance covered (m)	1605 ± 313
Farley et al. [25]	N: 10 (age: ND; Stature: ND; Weight: ND) S: Male C: Australia	ND	ND	During 20 min heats	SurfTraX, Southport, Australia	10	ND	Placed in a sealed arm strap and tightened around the bicep	Total distance (paddling + wave riding) (m)	997 (628–1678)
									Average speed per wave (km/h)	16.7
									Peak wave ridings speeds (km/h)	25.2 (19–31)
									Max distance covered during wave (m)	132 (82–180)
Farley et al. [26]	N: 41 (age: 23.2 ± 6.1 y; Stature: 177.2 ± 6.4 cm; Weight: 71 ± 10.3 kg) S: male C: Australia	PRO	3 professional domestic surfing events	Heats of 20 min	SurfTraX, Southport, Australia	10	ND	Placed in a sealed arm strap and tightened around the bicep	Wave count (n)	2.2.–7.7
									Max speed (km/h)	18.5–28.3
									Average speed (km/h)	16.6–20.2
									Total wave distance (m)	24–117
									Between-wave distance (m)	650–1008

**Note:** GPS: global positioning system; ND: not detailed; PRO: professionals; min: minimum; max: maximum.

**Table 4:** Surfing demands during training sessions.

Ref.	Sample				Device’s Characteristics				Results	
	Participants	Level	Context	Duration	Device Model	Hz	Number of Satellites Used	Place	Variable	Value
Barlow et al. [22]	N: 39 (age: 24.5 ± 6.3 y; Stature: 1.77 ± 0.12 cm; Weight: 72.6 ± 9.9 kg) S: Male C: United Kingdom	Recreational	Training	60 sessions	Polar G3 monitor (Polar Electro, Oy, Finland) Device	1	ND	*dry-bag*	N of rides per h	20.6 ± 11.4
									Max of ride speeds (m/s)	6.1 ± 1.2
									Mean ride time	13.0 ± 5.0 (min: 4.7 ± 1.5; max: 27.3 ± 13.3)
									Mean ride distance (m)	54.8 ± 25.4 (min: 16.5 ± 7.3; max: 117.7 ± 63.4)
									S of ride distances (m)	32.0 ± 18.8
									Total distance whilst surfing (%)	25.6 ± 9.6
									Total distance whilst surfing per h	891.4 ± 378.9
									Total time waiting (%)	41.8 ± 9.8
									Total time waiting per h	1452.9 ± 440.7
									Total time padding (%)	47.0 ± 6.1
									Total time paddling per h	1636.6 ± 374.8
									Total time riding (%)	8.1 ± 5.3
									Total time riding per h (s)	282.6 ± 195.2
									Total time miscellaneous (%)	3.1 ± 1.9
									Total time miscellaneous per h (s)	107.9 ± 69.2
									Total distance per h (s)	3925.5 ± 1239.8
									Average speed (m/s)	4.2 ± 1.1
Silva et al. [27]	N: 12 (age: 16 ± 1 y; Stature: 166.65 ± 8.25 cm; Weight: 58.15 ± 8.19 kg) S: 6 male and 6 female C: Portugal	Junior	Training	36 training sessions	Polar V800, Polar Electro, Kempele, Finland	2.4	ND	ND	Duration (min)	46.07 ± 5.23
									Total distance (m)	3188.75 ± 402.83
									Average speed (km/h)	4.16 ± 0.45
									Max speed (km/h)	14.94 ± 2.52
									Average pace (m/min)	69.48 ± 7.50
O´Neill et al. [28]	N: 10 (age: 33.20 y; Stature: 179.21 ± 5.07 cm; Weight: 81.91 ± 10.08 kg) S: male C: Australia	Recreational	Training	A 2 h session	GPSports HPISPU	15	ND	sports vest was fitted over the top	Average distance (m)	4974.18 ± 542.62
									Average speed (km/h)	2.48 ± 0.27
									Peak speed (km/h	31.86 ± 3.51
Secomb et al. [29]	N: 15 (age: 22.1 ± 3.9 y; Stature: 175.4 ± 6.4 cm; Weight: 72.5 ± 7.7 kg) S: male C: Australia	Amateur	Competition	2 h	VX Sport VX110 Log, Visuallex Sport International Ltd., Lower Hutt, New Zealand	4	ND	ND	Distance covered (m)	6293.2 ± 1826.1
									Average speed (m/min)	52.4 ± 15.2
									Time spent paddling (%)	42.6 ± 9.9
									Time spent sprint paddling to catch waves (%)	4.1 ± 1.2
									Time spent stationary (%)	52.8 ± 12.4
									Time spent wave riding (%)	2.5 ± 1.9
									Recovery of the surfboard (%)	2.1 ± 1.7

**Note:** GPS: global positioning system; ND: not detailed; PRO: professionals; min: minimum; max: maximum.

## Data Availability

Not applicable.
